# A Fast and Accessible Methodology for Micro-Patterning Cells on Standard Culture Substrates Using Parafilm™ Inserts

**DOI:** 10.1371/journal.pone.0020909

**Published:** 2011-06-07

**Authors:** Sahar Javaherian, Kylie A. O’Donnell, Alison P. McGuigan

**Affiliations:** 1 Department of Chemical Engineering and Applied Chemistry, University of Toronto, Toronto, Ontario, Canada; 2 Institute of Biomaterials and Biomedical Engineering, University of Toronto, Toronto, Ontario, Canada; University of Minho, Portugal

## Abstract

Micropatterning techniques provide direct control over the spatial organization of cells at the sub-mm scale. Regulation of these spatial parameters is important for controlling cell fate and cell function. While micropatterning has proved a powerful technique for understanding the impact of cell organization on cell behaviour, current methods for micropatterning cells require complex, specialized equipment that is not readily accessible in most biological and bioengineering laboratories. In addition, currently available methods require significant protocol optimization to ensure reliable and reproducible patterning. The inaccessibility of current methods has severely limited the widespread use of micropatterning as a tool in both biology and tissue engineering laboratories. Here we present a simple, cheap, and fast method to micropattern mammalian cells into stripes and circular patterns using Parafilm™, a common material found in most biology and bioengineering laboratories. Our method does not require any specialized equipment and does not require significant method optimization to ensure reproducible patterning. Although our method is limited to simple patterns, these geometries are sufficient for addressing a wide range of biological problems. Specifically, we demonstrate i) that using our Parafilm™ insert method we can pattern and co-pattern ARPE-19 and MDCK epithelial cells into circular and stripe micropatterns in tissue culture polystyrene (TCPS) wells and on glass slides, ii) that we can contain cells in the desired patterns for more than one month and iii) that upon removal of the Parafilm™ insert we can release the cells from the containment pattern and allow cell migration outward from the original pattern. We also demonstrate that we can exploit this confinement release feature to conduct an epithelial cell wound healing assay. This novel micropatterning method provides a reliable and accessible tool with the flexibility to address a wide range of biological and engineering problems that require control over the spatial and temporal organization of cells.

## Introduction

Micropatterning techniques to control the spatial organization of cells at the sub-mm scale are useful for tissue engineering [1], biosensor technology development [2], and for asking fundamental questions about the dependence of cell behaviour on local tissue organization [3], [4]. Micropatterning techniques provide direct control over several spatial parameters including colony or cell sheet size, distance between colonies, and with some methods, homotypic or heterotypic cell–cell contact [5], [6]. Regulation of these spatial parameters is important for controlling cell fate and cell function [7]. For example, the size and spacing of human embryonic stem cell colonies influences the differentiation trajectory of the cells [6], [8] and the presence of heterotypic cell-cell contact between hepatocytes and stromal cells improves maintenance of the hepatocellular phenotype *in vitro* [9]. While micropatterning has proved a powerful technique for understanding the impact of cell organization on cell behaviour, current methods for micropatterning cells such as dielectrophoresis [10], microfluidic patterning [11], micro-contact printing [7], and ink-jet microprinting [12] all require complex, specialized equipment that is not readily accessible in most biological and bioengineering laboratories. Furthermore, many of these methods require significant protocol optimization to ensure reliable and reproducible patterning. The inaccessibility of current methods has severely limited the widespread use of micropatterning as a tool in both biology and tissue engineering laboratories. Here we present a simple, cheap, and fast method to micropattern mammalian cells into stripes and circular patterns using Parafilm™, a common material found in most biology and bioengineering laboratories. Our method does not require any specialized equipment and does not require significant method optimization to ensure reproducible patterning and although our method is limited to stripe and circular patterns these geometries are sufficient for conducting experiments to address a wide range of biological problems.

Currently the simplest and most common methods for micropatterning cells are microcontact printing [4], microfluidic patterning [11], and the use of microstencils [13], [14]. Each of these methods utilizes an elastomeric stamp or membrane, usually made of PDMS, prepared by casting the liquid prepolymer of an elastomer against a master with a patterned relief structure, fabricated using photolithography [4]. Cell adhesive and non-adhesive regions are then created on a substrate (usually glass, polystyrene or tissue culture polystyrene (TCPS)) by physically blocking specific regions of a cell-adhesive substrate using the stamp or membrane (in the case of microfluidic or microstencil patterning) or by selectively depositing cell adhesive proteins in specific regions of a cell-repellent substrate (in the case of microcontact printing). The major limitations of these methods include i) a clean room is required to generate the original topographic master for stamp or channel fabrication, ii) patterning, particularly using microcontact printing, is often variable and significant protocol optimization is required to ensure the technique is reproducible, iii) in the case of microstencils and microcontact printing, the cells are confined to the patterns for only approximately three to four days [15], after which they deposit enough ECM to migrate to all positions on the substrate, iv) only microfluidic and some microstencil methods allow release of the cells from the patterns deliberately at a specific time, v) generating patterned co-cultures is not possible for all methods and in the case of microcontact printing requires the use of serum-free medium [1] and vi) many of the methods have not been adapted for use with 96-well plates, which are the most common substrates used for biological studies.

Currently, no cell patterning method exists that combines all of the following features: i) extreme simplicity and easy implementation, ii) no need for access to any specialized instrumentation/technology for generation of the cell patterns, iii) low cost, iv) the ability to produce patterns that can be maintained for an undefined amount of time and released on-demand, v) the capacity to facilitate a co-culture of different cell types and vi) compatibility with 96-well plates. We set out to develop a micropatterning method with all of these features, which we believe will provide a reliable and accessible tool with the flexibility to address a wide range of biological and engineering problems that require control over the spatial and temporal organization of cells.

Here we present a method that utilizes Parafilm™ inserts to spatially restrict cell adhesion to the underlying substrate. We selected Parafilm™ as an attractive option for cell patterning because it is i) commercially available and present in most laboratories, ii) cheap, iii) non-toxic [16] and therefore will not affect cell viability during the patterning procedure, iv) easy to handle and cut into a desired pattern, even when handling small pieces, v) cell repellent and therefore does not allow cell growth or adhesion, and vi) self-adheres reversibly when pressed down firmly on a TCPS or glass surface allowing easy removal of the Parafilm™ inserts at any time after cell patterning. We demonstrate that using Parafilm™ inserts we can pattern a number of cell types (ARPE-19 epithelial cells and MDCK epithelial cells) into circular and stripe patterns in TCPS wells and on glass slides (two common culture substrates) and that we can contain cells in the desired patterns for more than one month. Furthermore, since we can easily remove the Parafilm™ insert we also show that we can use our method to generate co-culture patterns and to release the cells from the containment pattern and allow cell migration outward from the original pattern. Finally, we exploit this confinement release feature of our method to conduct an epithelial cell wound healing assay, which is challenging to conduct using a traditional wounding method because wound generation tends to tear large sections of the epithelial cell sheet or cause significant damage to the cells at the wound edge [13]. This cellular damage causes difficulties in distinguishing the effect of cell damage versus the presence of open space on cell migration behaviour.

To our knowledge, our Parafilm™ patterning method is the first example of a patterning technique that can control cell organization down to dimensions of 150 µm while not requiring the use of a clean room. Our method is accessible since it required only needles, razor blades and Parafilm™ to generate the patterning inserts and therefore can be more easily adopted by laboratories without microfabrication experience and without access to complex fabrication tools. Furthermore, our method is flexible for studying cell behaviours in isolated colonies, in co-culture, and during dynamic cell re-organization, for example during wound healing or collective cell migration.

## Methods

### Parafilm Patterning strategy

**Figure 1:**
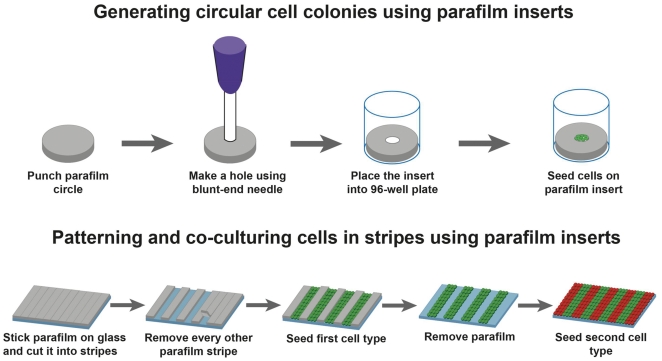
Schematic representation of Parafilm™ insert cell patterning method. *Top panel* – Fabrication of Parafilm™ inserts for generation of circular cell patterns. Parafilm™ is cut into circles using a biopsy punch and holes of desired size are generated using a blunt-ended needle. The Parafilm™ insert is placed into a well of a 96-well plate and seeded with cells. Cells only grow on TCPS not covered with Parafilm™. *Bottom panel* - Fabrication of Parafilm™ inserts for generation of stripe cell patterns and co-cultures. A piece of Parafilm™ is placed on a glass coverslip and cut into stripes using a surgical blade. Every other parafilm stripe is removed to expose the underlying glass. The first cell type is seeded and cells only adhere to the exposed stripes of glass generating a stripe patterns. Once cells are attached, the remaining stripes of Parafilm™ are removed and the second cell type seeded.

Figure 1 outlines our strategy for patterning cells using Parafilm™ inserts. To generate inserts that fit into wells of a 96-well plate we cut circular pieces of Parafilm™ M (Pechiney Plastic Packaging Company, USA) using a 6 mm diameter biopsy punch (Frey Products Corp., Buffalo, NY). To obtain the circular holes required for cell patterning, we had to develop a method to generate reproducible holes in the Parafilm™ inserts. Puncturing Parafilm™ with a small sharp tool such as a needle produces outward deformation of the Parafilm™ generating a raised lip as opposed to a clean hole with a reproducible size. We therefore generated holes using fine gauge blunt-ended tip needles, which ensured we cut a hole with predictable dimensions out of the Parafilm™, without deformation of the film into a raised lip. It was also important to cut the holes on a hard surface, such as glass, to further reduce unwanted deformation of the Parafilm™ during generation of the hole. We used 26 G and 30 G needles (Ameritronics, CA), which have reported inner diameters of 220 µm and 150 µm, respectively. Once cut to size we pressed the Parafilm™ insert firmly into the well of a 96-well plate for cell patterning.

To generate a stripe as opposed to a circular pattern we cut out a 20 mm ×20 mm square of Parafilm™ using a scalpel and pressed it tightly on top of a 22 mm ×22 mm glass cover slip (VWR, Canada). We made incisions in the Parafilm™ using two surgical blades (No 10, Feather, Japan) taped together to ensure a uniform width of the stripes. We then removed every other stripe of Parafilm™ using tweezers, producing a glass coverslip covered with 400 µm-wide stripes of Parafilm™ separated by a distance of 300 µm. To generate larger spaced stripes we inserted spacers of set thickness between the two blades before taping.

Before using the inserts for cell culture, we added phosphate buffer saline (PBS) ensuring that the Parafilm™ patterns were completely submerged in PBS and degassed the films for 5 minutes at a pressure of 30 psig. This step was critical to ensure liquid infiltration into the small holes or stripes within the insert and subsequent cell patterning. We UV sterilized the films in PBS for 30 minutes and then removed the PBS before cell culture. For all cultures on glass substrates we also incubated the membranes with FBS (Sigma Aldrich, Canada) at 37°C for one hour prior to seeding of cells to ensure good cell adhesion to the glass.

### Cell Culture

We used the ARPE-19 human retinal epithelial cell line (ATTC, Manassas, VA**)** and the MDCK dog kidney epithelial cell line (ATTC) at passages between P10-20. For co-patterning experiments we used ARPE-19 cells infected with a lentivirus encoding GFP. MDCK cells were grown in Dulbecco’s modified Eagle’s medium (DMEM, BioWhittaker, Canada) supplemented with 10% FBS and 1% Penicillin/Streptomycin (VWR, Canada). ARPE-19 and MDCK/ARPE-19 co-patterned cultures were maintained in DMEM/F-12 (Invitrogen, Canada) supplemented with 10% FBS and 1% Penicillin/Streptomycin. All cell cultures were maintained in an incubator at 5% CO_2_.

### Cell seeding into the patterns

To seed cells on the circular patterns (150 µm diameter) in 96-well plates we placed droplets containing 1000 cells onto the holes in the Parafilm insert. We left this in the incubator for 1 hr to allow cells to stick down within the holes before lightly washing once with PBS to remove un-adhered cells. We then added 100 µL of DMEM/F-12 into each well. Cells were cultured on the micropatterns for desired time periods and the culture medium changed every three days. To seed cells on the stripe patterns we removed the FBS pre-wash (done for all glass substrates) and applied a cell suspension of 2×10^6^ cells/mL making sure that the entire surface of the glass coverslip was covered by the cell suspension. We incubated the samples at 37°C for 3 hours and then gently washed away any un-adhered cells with fresh culture medium. After an overnight incubation we washed the patterned cells three times with fresh medium, and continued the culture for up to 4 weeks periodically supplying the cells with fresh medium.

### Assessing cell viability, containment, and release from the patterns

Using light microscopy we imaged patterned cell colonies at days 1, 3, 7, and 14. At each time point we assessed cell morphology (did the cells look viable) and containment within the pattern (did the cells migrate on to the Parafilm™ insert). To determine the viability of cells for the duration of their containment, we stained cells with Trypan blue (2 min wash with Trypan blue and then assessed for number of dead (blue) cells). To assess how well cells could be released from containment we removed the Parafilm™ insert using tweezers after day 7 of culture and took images of the cell patterns immediately after removal and 24 hours following removal of the insert.

### Co-culture cell patterns

We performed co-patterning using both circular and stripe Parafilm™ inserts. We seeded MDCK cells in circles or ARPE-19-GFP cells on stripes as described above and cultured the patterned cells overnight. The next day, we removed the Parafilm™ insert using tweezers and incubated the patterned cells in FBS for 20 minutes. We next seeded a second cell type (ARPE-19) at a seeding density of 3×10^6^ cells/mL and incubated for one hour to allow the additional cells to adhere to the surface free from the first cell type. We then thoroughly washed the substrates with growth medium to remove any un-adhered cells and cultured the co-culture patterned cells overnight or for 7 days. We stained co-cultures generated in stripe patterns with DAPI, and imaged using light and fluorescence microscopy to show the distribution of the two cells types on the substrates over time.

### Epithelial wound healing experiments

We seeded ARPE-19 cells in a striped pattern as described above and cultured them overnight. We then removed the parafilm insert and allowed the cells to repopulate the area between the striped pattern for 1, 4, 10 or 18 hours. During this time period the cells were cultured in DMEM/F12. After the desired period of migration we fixed the cells in 4% paraformaldehyde, washed in phosphate buffered saline, permeabilized using 0.1% tween and stained with DAPI (Invitrogen, Canada) and phalloidin (F-actin staining, Invitrogen, Canada) for fluorescent microscopy.

### Substrate cell migration assessment

Parafilm inserts for 96 well plates were prepared (see above). The inserts were placed into the wells and pressed against the well floor to promote the bonding between TCPS and the parafilm. We left the parafilm inserts adhered to TCPS overnight. We next removed the parafilm inserts and seeded 8×10^3^ ARPE19 cells on four different groups of surfaces: 1 – unmodified TCPS, 2 – TCPS blocked with FBS for 1 h at 37°C, 3 – TCPS after removal of parafilm inserts, and 4 – TCPS after removal of parafilm inserts and blocking with FBS for 1 h at 37°C. The cells were left to adhere to the substrates overnight. Next, we stained the cell nuclei with 500 ng/mL Hoechst 33342 (Invitrogen) for 30 min at 37°C. We next monitored cell migration by collecting images at 30 min intervals in an Image Express Micro high content screening system (Molecular Devices, Sunnyvale, CA). Cell velocity was determined by tracking the positions of cell nuclei using the Metamorph cell tracking algorithm. We used a one-way ANOVA test to compare the mean velocities of cells in the four categories (n = 5) with p-values of <0.01 considered significant. Error bars represent standard deviations.

## Results

### Parafilm™ insert fabrication

We generated circular patterns in the Parafilm™ (Supplementary information Figure S1) using fine gauge blunt 26 G and 30 G needles (diameters of 220 µm and 150 µm, respectively). We were able to obtain holes with high precision and high homogeneity in size distribution. For the 26 G needle we obtained holes with a mean diameter of 219.3±8.2 µm (± standard deviation where n = 15) and for the 30 G needle we obtained holes with a mean diameter of 149.8±8.0 µm (± standard deviation for n = 15). Needles could be used to make approximately 100 holes before becoming too dull (or distorted in any way from the pressure of repeated use) to produce reproducible holes with circular geometry (Table S1, supplementary information). To generate stripe patterned Parafilm™ we used two razor blades taped together and were able to obtain striped holes in the Parafilm™ of 300.3±21.2 µm (± standard deviation for n = 10) in size. We also generated larger stripes by placing a spacer between the two blades. Using scotch tape and two overlaid pieces of laboratory tape as spacers we obtained striped holes in the Parafilm™ of 370.6±18.5 µm and 551.2±16.9 µm (± standard deviation for n = 10) in size, respectively.

To ensure acceptable bonding between the TCPS or glass substrate and the Parafilm™ insert it is crucial to apply significant pressure to the insert, while making sure that the Parafilm™ insert is not deformed or stretched. The bonding between parafilm and the underlying surface is solely facilitated by reversible adhesion of parafilm to glass and TCPS (i.e. no glue is used). We used a wooden rod with a rolling pin action over the parafilm insert before and after removal of stripes to ensure strong bonding. In the case of the 96-well plate insert we fabricated a 6 mm-diameter press made of PMDS and used it to push the parafilm insert onto the bottom of each well. Additionally, we found the step of degasing was a good test for the strength of the bond between Parafilm™ and glass. If the bond is too weak or leaky, the Parafilm™ inserts detach from the surface during degasing.

### Cell patterning, viability and containment

**Figure 2:**
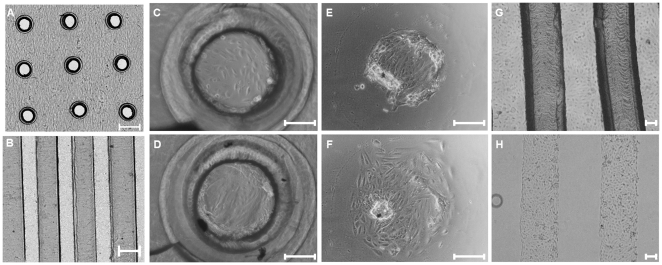
ARPE-19 cells contained in circular and stripe patterns. (A) Cells 1 day after seeding into holes in Parafilm™ generated using 26G needle. The image was generated by taking sixteen images at 4X magnification and stitching them together. (B) Cells 1 day after seeding into stripes in Parafilm™ generated using two blades taped together. The image was generated by taking twelve images at 4X magnification and stitching them together. (C) Cells 1 day after seeding into holes in Parafilm™ generated using 26G needle. (D) Cells 7 days after seeding in circular pattern. (E) Cells (after 7 days of containment) immediately after Parafilm™ insert removal. (F) Cells imaged 24 hours after the removal of the circular Parafilm™ insert. Over the 24 h period the cells spread outwards from original patterned area. (G) Cells 1 day after seeding on parafilm inserts with stripes pattern. (H) Cells imaged immediately after removal of parafilm insert. The scale bar is 300-µm wide for A and B and 100-µm wide for C,D,E,F,G, and H.

Cells seeded in wells containing Parafilm™ inserts with circular holes formed circular patterns by day 1 (Figure 2A,C and supplemental information Figure S2 and S3). The cells remained contained within the patterns at 7 days (Figure 2D). Over time the cells became confluent and covered the entire pattern. As time increased further and cells became post-confluent within the patterned holes, they started to aggregate at the side of the Parafilm™ walls but did not outgrow the patterns or migrate on to the Parafilm™. No cells stained positive for Trypan blue after seven days of confinement indicating that cells remained viable in the Parafilm™ inserts (Supplemental information Figure S3). Furthermore, when the Parafilm™ insert was removed at day 7 cells migrated outward from the original circular containment region (Figures 2E, F and Supplemental information Figure S2D) indicating that the cells were viable and capable of normal migration. Similarly, cells seeded on the stripes patterns of Parafilm™ were strictly contained to the area free of Parafilm™ (Figure 2B, G,H, supplemental information Figure S4). They did not grow over the Parafilm™ even after 4 weeks in culture (results not shown). By removing the Parafilm™ inserts, we obtained stripe patterns of confluent cells (Figure 2H). In the case of the stripes on glass substrates (as opposed to TCPS), we found that attachment of the cells to the glass was greatly enhanced by treating the glass with FBS or fibronectin (if serum in the culture is undesirable) for one hour prior to seeding the cells.

### Patterned Co-Cultures

**Figure 3:**
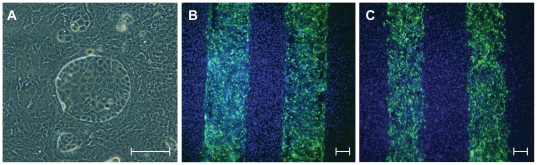
Generation of co-culture of two cell types using parafilm patterning. (A) MDCK circular colonies were obtained by culturing the cells in a circular pattern. A second cell type (ARPE-19) was seeded resulting in a co-culture of the two cells types in a circular pattern. (B and C) GFP-labelled ARPE-19 cells were cultured in stripes overnight. A second cell type (non-labelled ARPE-19) was seeded. Cells were then co-cultured overnight (B) or for one week (C) before imaging with fluorescence microscopy. In (B) and (C) *Green* indicates GFP and *blue* indicates dapi (cell nuclei). The scale bar for each image is 100-µm wide.

We wanted to test the ability of our method to generate patterned co-cultures since this is particularly challenging using currently available patterning methods, and is useful for studying the interactions between different constituent cells in a tissue. We co-cultured two cell types using both circular and stripe insert patterns (Figure 3). Co-culture patterns were maintained in the epithelial cell cultures for one week. Figure 3 shows that even after one week in a culture the stripe co-culture remained intact without significant disturbances to the original pattern (Figure 3B and C). We found that an additional 20-minute FBS or fibronectin treatment between removal of the Parafilm™ insert and seeding of the second cell type greatly increased the attachment of the cells making it easier to preserve the desired pattern. We speculate that proteins present in the FBS wash adsorb to the substrate and mask any hydrophobic cell-repellent residue left behind from the Parafilm™ making the surface more cell-adhesive. Additionally, we found that the initial cell seeding density is very important for obtaining precise pattern features. For the epithelial cell types we used in this study, seeding using 2 million cells/mL for the first cell type and 3 million cells/mL for the second cell type was optimal. However, it is possible that the optimal cell density is different for other cell types depending on their size, adhesiveness to the substrate and tendency to form aggregates.

To ensure sharp pattern features it was important to remove the parafilm insert by slowly peeling it off from one side to the other as opposed to simply lifting it up (for example disruption of the pattern can be seen in Figure 2E). This was particularly important if the cells have been contained in the pattern for a number of days and are post-confluent. Additionally, we found that the presence of medium at the time of parafilm removal helps to prevent damaging the cell pattern.

Similar to all the other major co-patterning techniques currently available [5], [18] our technique allows generation of patterned co-cultures by treating the surfaces that the two cell types grow on differently. Accordingly, there is a possibility that the surface brought into contact with parafilm is slightly different from that of plain glass or TCPS. To address this we compared the cell migratory behaviour on these differentially treated surfaces (Supplemental information Figure S5) and saw no significant differences in cell migration behaviour between them (p = 0.853).

### Epithelial cells wound healing assay

**Figure 4:**
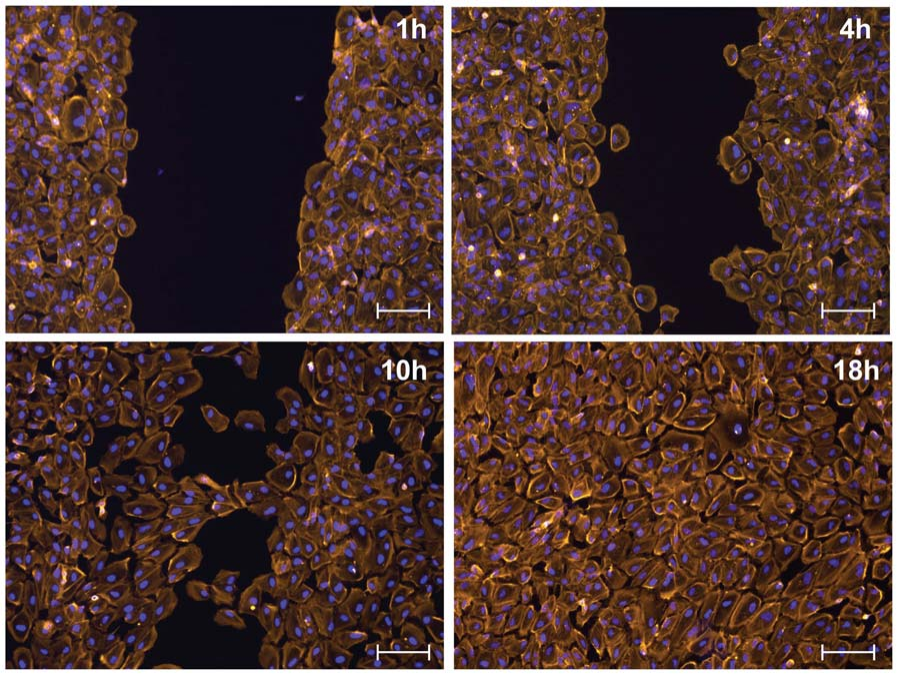
Fluorescence microscopy images of epithelial cells after removal of Parafilm™ inserts: Epithelial cells (ARPE-19) were cultured in stripe patterns. The Parafilm™ stripes were removed enabling cells to re-populate the free surface created by removal of the parafilm. Cells were fixed at 1,4, 10 and 18 hours after insert removal and stained with DAPI (*blue*) to show the nucleus and with phalloidin *(orange)* to show the F-actin cytoskeleton. The scale bar for each image is 100-µm wide.

We wanted to demonstrate the utility of our method for releasing cells from confined patterns at a desired time point, since this feature is also not commonly possible using currently available patterning strategies. A simple example application of cell release from a confined pattern is a wound-healing assay. Unlike conventional scratch wound healing assays [17] however, our method does not result in significant cellular damage of the cell sheet at the wound edge. Figure 4 shows images of patterned attached epithelial cell sheets at different times after removal of the parafilm insert. The attached epithelial cell sheet migrated into the open space created by removal of the Parafilm™ insert. Cells from opposite sides of the “wound” formed bridges across the “wound” and eventually filled the entire wound region by 18 h after release from the pattern. By controlling the width of the Parafilm™ insert between cell stripes we were also able to control “wound” width (Supplementary information Figure S4).

## Discussion

We set out to develop a reliable and widely accessible micropatterning tool with the flexibility to address a wide range of biological and engineering problems that require control over the spatial organization of cells. Specifically, we wanted to design a method that i) would be extremely simple and compatible with 96 well plates and glass slides, ii) that did not require access to specialized equipment, iii) that could maintain cell patterns for an undefined amount of time and iv) that allowed release of the cells from these patterns on demand providing the capacity to facilitate cell migration studies or co-culture of different cell types. The novel Parafilm™ insert patterning technique described here has all these desirable features. Our method is extremely simple, cheap and compatible with standard cell culture substrates. It requires significantly less time to perform (3–4 h) than microcontact printing, which is currently the most common patterning strategy and can take up to a day to generate the seeded cell patterns (not including generation of the photolithographic master and microstamp). Furthermore, we could pattern and contain cells for more than a month, which is significantly longer than possible with conventional methods. We also demonstrated that we could release cells from the patterns on demand by removal of the Parafilm™ containment insert. This facilitated patterned co-culture and cell migration experiments. Here we present two ways of cutting parafilm that allow generation of circular and striped patterns. This limits the possible geometries of cell colonies to circles and stripes, however these geometries are sufficient for addressing a wide range of problems requiring spatially organized cell populations.

Our novel method uses Parafilm™ to contain cells while culturing them on TCPS or glass and involves cutting patterns out of Parafilm™ sheets using needles and blades to produce circular and stripe patterns respectively. While, here, we only present applications of parafilm inserts for cells cultured on polystyrene and glass, we foresee that the method could be adapted for use with other sturdy supports. The use of this method with other supports requires that the parafilm insert can be firmly pressed against the surface to promote good adhesion between the parafilm insert and the substrate surface. As such, it is unlikely that parafilm inserts could be used to pattern cells on hydrogel surfaces. Additionally, the surface must allow easy removal of entrapped gas bubbles within the holes of the parafilm insert, as failure to do so results in inability of cells to enter the holes in the parafilm.

In the case of circular pattern generation, the range of needle inner diameters (and hence hole and pattern diameters) commercially available ranges from 3.8 mm to 80 µm respectively (Product data from ameritronics.com) therefore our method can potentially pattern circular colonies up to 3.8 mm and down to 80 µm in diameter, although we only demonstrated dimensions of 220 µm and 150 µm here. Spatially controlling cell organization at the size scales possible using these commercially available needles is appropriate for addressing problems that currently use less accessible microstamping techniques. For example, colonies of human embryonic stem cell with dimensions between 100 and 500 µm produced different levels of endogenous Jak-Stat signalling pathway activation (8).

In the case of stripe inserts, we can achieve a range of stripe widths based on the spacing of two parallel razor blades. The smallest stripe size achievable was 300 µm in width, which corresponded to two blades taped together with no intermediate spacer. We demonstrate generation of stripes of different width by placing spacers between the two blades (Supplementary information Figure S4); the width can be adjusted by using spacers with a specific desired thickness. The pattern dimensions, feasible using the blade cutting system, are also within a relevant size scale for asking questions about the effect of cellular organization on cell behaviour. For example, Krupffer cells co-cultured with hepatocytes in stripes of 500 µm drastically improved the hepatocyte function compared to a monoculture [18]. This size scale is also commonly used in wound healing assays [13], [17].

The two major limitations of our method are that we can only generate circular or stripe geometry and that these patterns must have dimensions greater than 150 µm. Our patterning method is therefore unsuitable for single cell patterning applications where single cells can be patterned into a specific geometry [14]. While not suitable for single cell dimension, our method is expected to be very useful in studies of cellular interactions where such small dimensions are not required [8], [13], [18], [19]. Alternatives to using circular needle punches and blades to obtain non-circular or stripe holes could however be explored in the future to expand this method for more diverse geometries and pattern sizes. Our main goal here however was to make the method as accessible and simple as possible to facilitate patterning, albeit at the 150 micron-scale, in any biology or bioengineering lab. Additionally, we recognize that as with existing co-culture patterning techniques, our parafilm-based method results in the two patterned cell populations being grown on slightly different surfaces since the TCPS or glass surface could in theory be modified by any remnant residue of parafilm after insert removal. Although we blocked the surface with FBS prior to both cell seeding steps to negate these possible differences, we cannot rule out that the surfaces are slightly different. We did find however, that cell migration on TCPS brought in contact with parafilm is not different from that of TCPS that has not contacted Parafilm, suggesting that any slight differences in these surfaces has a minimal effect, if any, on the cell behaviour.

A key feature of our method is the ease with which the Parafilm™ insert can be handled and removed at any desired time point. It is therefore possible to maintain the cells in a pattern for a desired period of time and then remove the Parafilm™ insert to either “release” the cells or provide a culture substrate for a second cell type. Using our patterning and release system we conducted a wound closure assay on attached epithelial cell sheets. We selected this as an example experiment to demonstrate the potential utility of our system for conducting common biological experiments that are currently challenging under some circumstances. Wound closure assays, while used regularly to study endothelial cell migration, are difficult to conduct on epithelial cells since the strong adhesion between epithelial cells can result in tearing of the cell sheet, making it difficult to generate a clean wound and distinguish between the effects of open space versus cell damage on the resulting cell migration behaviour [13]. The results we obtained using our injury-free wound healing assay suggest that the presence of true injury in cells bordering the “wound” is not necessary (introduction of free space is sufficient) to induce migration of epithelial cells, consistent with the literature [13], [19]. Commercial systems, such as the Ibidi insert system, exist for conducting epithelial wound closure experiments however Ibidi inserts are expensive and come in limited geometries with a set “wound” width. Our method enabled the study of epithelial wound closure at a fraction of the cost of using Ibidi culture-inserts and allowed flexibility in the size of the “wound” we wished to generate. This provides just one simple example of how our Parafilm insert method could be used to conduct a common biological assay. We anticipate the simplicity of our method will provide a valuable tool for applications in a range of biological problems requiring spatial control of cell organization.

### Conclusion

We have developed a simple method to pattern cell organization on standard cell culture substrates. Our method is more accessible than currently available techniques because it does not require the use of a clean room or photolithography. Cells can be contained in the desired pattern for at least a month and released from pattern containment on demand. Easy removal of the Parafilm™ insert facilitates co-culture cell patterning and cell migration experiments. The ease, speed and flexibility of our method makes it a useful technique that can be employed by any research group to characterize cell behaviour under spatially controlled culture conditions ending inaccessibility of micro-patterning methods to non-engineering research groups.

## Supporting Information

Figure S1 Light microscopy images of holes generated in Parafilm inserts using blunt needles. (A) Hole created by 26G needle resulted in a hole diameter of 220 µm. (B) Hole created by 30G needle resulted in a hole diameter of 150 µm. The scale bar for each image is 100-µm wide.
(TIF)

Figure S2 Light microscopy images of patterned cells using inserts created with 30G needles. (A) Cells at day 1 after seeding. (B) Cells at day 7 after seeding. (C) Cells contained for seven days immediately after the Parafilm insert has been removed. (D) Cells imaged 24 hours after the removal of the Parafilm insert. Cells spread and migrate outwards from original patterned area. The scale bar for each image is 100-µm wide.
(TIF)

Figure S3 Light microscopy images of cells patterned with inserts created using a 30 G needle and stained with trypan blue to assess cell viability. (A) Cells at day 7 with Parafilm insert in place. (B) Patterned cells contained for day 7 after the Parafilm insert has been removed. (C) Patterned cells contained for 7 days stained with trypan blue. No blue cells were visible indicating all cells in the pattern were viable. The scale bar for each image is 100-µm wide.
(TIF)

Figure S4 Light microscopy images of cells patterned with inserts created using two blades taped together with spacers in between to generate stripes of varying width. (A) Cells cultured with a parafilm insert generated using two blades spaced by 2 pieces of laboratory tape. (B) Cells cultured with a parafilm insert generated using two blades spaced by 1 piece of scotch tape. (C) Cells cultured with a parafilm insert generated using two blades taped together without a spacer. The scale bar for each image is 100-µm wide.
(TIF)

Figure S5 The effect of parafilm residue on cell migration. Cells were seeded on either plain tissue culture polystyrene, TCPS blocked with FBS, TCPS brought into contact with parafilm, and TCPS brought into contact with parafilm and blocked with FBS. Error bars shown represent standard deviation. An ANOVA test indicated no significant difference between any of the analysed groups (n = 5, p = 0.853).
(TIF)

Table S1 Accuracy of the shapes of the holes generated in parafilm inserts using blunt-ended needles. Since any inaccuracy in the generation of holes in parafilm using circular needles resulted in formation of ellipsoid holes (as opposed to circular ones), we measured the lengths of the minor and major axes of the generated holes to quantify the shape accuracy (n = 20). In the case of the perfect circle the ratio between the two axes equals one; the lower this ratio, the more distorted the shape of the generated holes.
(DOCX)
