# Supplementary material for: A Fast and Accessible Methodology for Micro-Patterning Cells on Standard Culture Substrates Using Parafilm™ Inserts
Source: PLoS One. 2011 Jun 7;6(6):e20909. doi: 10.1371/journal.pone.0020909 (PMC3110254; doi:10.1371/journal.pone.0020909)
Supplement: Table S1 — Accuracy of the shapes of the holes generated in parafilm inserts using blunt-ended needles. Since any inaccuracy in the generation of holes in parafilm using circular needles resulted in formation of ellipsoid holes (as opposed to circular ones), we measured the lengths of the minor and major axes of the generated holes to quantify the shape accuracy (n = 20). In the case of the perfect circle the ratio between the two axes equals one; the lower this ratio, the more distorted the shape of the generated holes. (DOCX) [file pone.0020909.s006.docx]

**Table S1.**

| **Needle size used to generate holes in parafilm** | **Average ratio between minor and major axes of the generated ellipse hole** | **Standard deviation** |
| --- | --- | --- |
| 30G | 0.982 | 0.063 |
| 26G | 0.985 | 0.050 |
